# Super-radiating manipulation of a nano-emitter by active toroidal metamaterials

**DOI:** 10.1038/srep46609

**Published:** 2017-05-09

**Authors:** Jie Li, Ming-Jie Zhu, Ying-Hua Wang, Ren-Chao Jin, Jia-Qi Li, Zheng-Gao Dong

**Affiliations:** 1 Physics Department and Key Laboratory of MEMS of the Ministry of Education, Southeast University, Nanjing 211189, China

## Abstract

The far-field radiation of a single dipolar emitter can be controlled by coupling to toroidal dipole resonance attached to metallic double flat rings, realizing a conversion from non- to super-radiating. The underlying physical mechanism is the hybridization interference of toroidal and electric dipoles under an asymmetric configuration by introducing a radial displacement of the dipolar emitter. By embedding gain medium in the gap spacer between double flat rings, the directional far-field super-radiating power can achieve a tremendous enhancement with a moderate requirement on the gain coefficient, promoting light-matter interaction manipulation.

As one member of the third family in electromagnetic multipole theory^1^,^2^, toroidal dipole was proposed by Zel’dovich in 1957 to interpret the parity violation on the weak interaction in nuclear and particle physics^3^, which is a result of current flowing on the surface of a torus along its meridian^4^,^5^. However, due to weakly coupling to electromagnetic radiation, there is an obstacle to observe the toroidal dipolar response usually masked by electric and magnetic multipoles^6^. Fortunately, the appearance of metamaterials laid a solid foundation for overcoming it. Metamaterials are artificially constructed and own sub-wavelength unit cells in periodic arrays^7^,^8^. In basis of its various novel electromagnetic properties unobtainable in naturally occurring systems, such as negative refraction, super-imaging, perfect absorber and cloaking^9^,^10^,^11^, metamaterials have been a hot topic of artificial electromagnetic materials. K. Marinov *et al*. in 2007 theoretically put forward a toroidal metamaterial to suppress electric and magnetic multipoles and strength the toroidal dipole resonance^12^. In recent years, more toroidal metastructures were designed to further explore optical properties of toroidal dipole either theoretically or experimentally, for example, double disks^6^,^13^, asymmetric double bars^14^, multifold double rings^15^, oligomer nanocavities^16^, and other metallic metastructures based on split ring resonators^5^,^17^,^18^. To further avoid ohmic-damping loss in metals, all-dielectric metamaterials were also proposed to realize high-Q-factor toroidal dipole resonance^19^,^20^.

In optical physics, light-radiation manipulation is always a hot area of research^21^,^22^,^23^,^24^. There are a lot of ways to manipulate light scattering, such as (i) by electric dipole resonance^25^, (ii) by optically-induced magnetic dipole resonance^26^,^27^, and (iii) by the interference between electric and magnetic dipole resonances, showing the forward directional scattering while suppressing the backward scattering^28^,^29^,^30^. With the new member of toroidal dipole joining, it brings an extra degree of freedom for light scattering manipulation^31^. For example, in our previous work, a dipolar emitter coupling to the metastructure by double flat rings not only excites toroidal dipole but also controls both radiating direction and power of the dipolar emitter by tuning geometric parameters, acting like a nanoantenna^13^. Furthermore, a four-U-shaped toroidal metastructure with optical gain medium was proposed by Huang *et al*. in 2013, obtaining a high-Q-factor toroidal resonance and realizing the so-called toroidal lasing spaser^5^. In this paper, we embed the gain medium into the gap spacer between metallic double flat rings to explore the influence of optical gain on the far-field scattering properties based on the coupling interference between toroidal and electric dipole radiations.

## Results and Discussion

**Figure 1:**
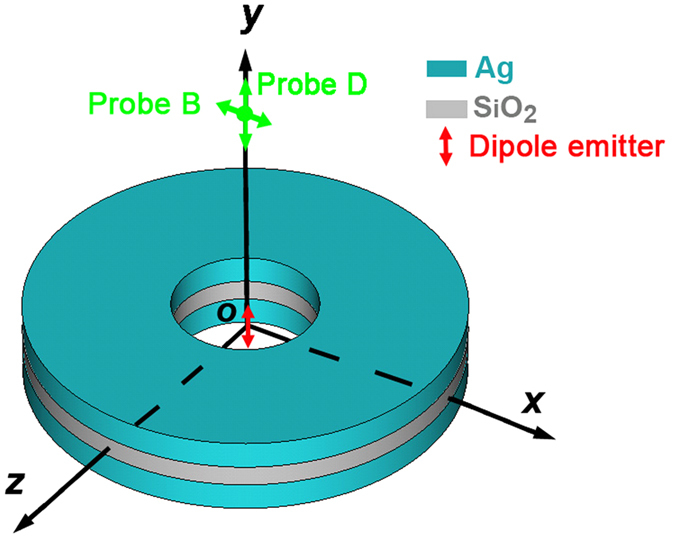
Schematic of the double-flat-ring toroidal metamaterial.

The double metallic flat rings metastructure is illustrated in Fig. 1. The metal is chosen as silver following the Drude-type dispersion model 
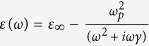
, with the high-frequency permittivity 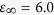, the plasma frequency 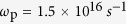 and the collision frequency 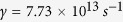. The outer and inner radii of 40-nm-thick silver rings are 430 nm and 100 nm, respectively. A 40-nm silicon-oxide (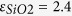) gap layer with gain medium sparsely embedded in was assumed to have a gain coefficient as 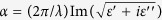, where 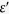 and 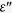 are accordingly real and imaginary parts of permittivity for the gain layer^5^. To probe the far-field optical radiating property, the far-field probes B and D (marked by green arrows) are located on the *y*-axis as shown in Fig. 1. A dipolar emitter (labeled by red arrow), along the *y*-axis with 1-nm length and oscillating electric current of 1 A, is chosen as the excitation source. Δ*x* is set as the radial displacement of the emitter with respect to the geometric center “*O*” of double-ring along the *x*-axis. Note that “*O*” is also the origin of coordinate system. The numerical simulation is performed based on the full-wave finite-element method (Ansoft HFSS).

**Figure 2:**
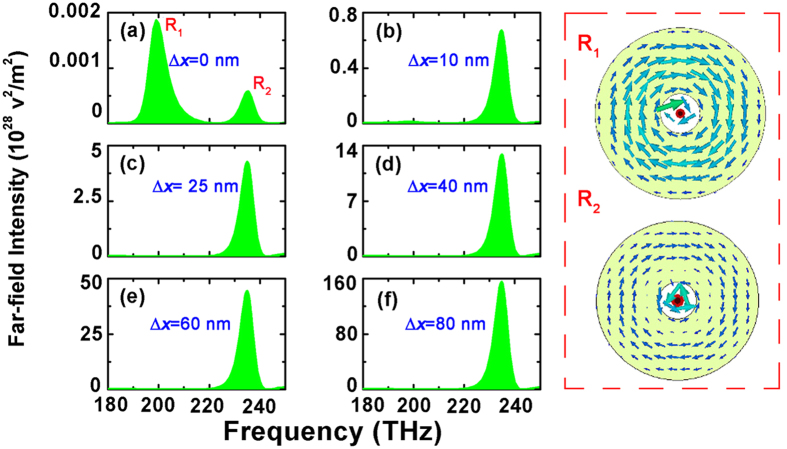
The spectra of far-field intensity monitored by probe B for different radial displacement Δ*x*. Inset maps in the right pink box are magnetic field distributions of constructive (resonance *R*_1_) and destructive (resonance *R*_2_) interferences between toroidal and electric dipoles at 200 THz and 235 THz, respectively.

**Figure 3:**
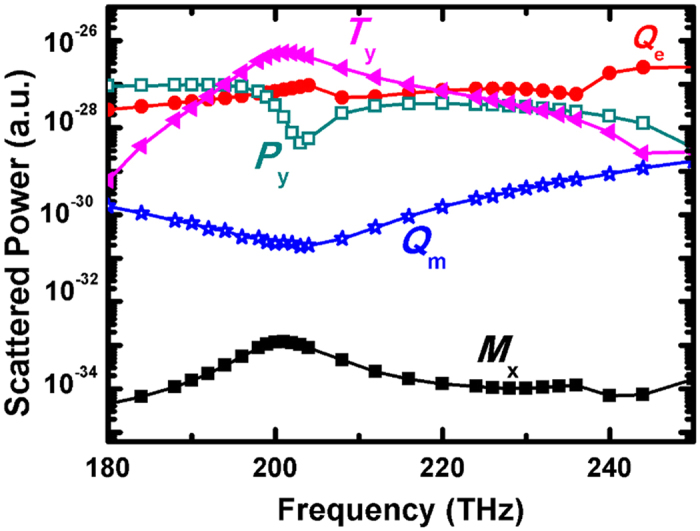
The scattered powers in terms of multipoles when Δ*x* = 0 nm.

In comparison to field probes in other directions^13^, the far-field radiating power, recorded by the probe B on the y-axis as shown in Fig. 1, is much appropriate for revealing the directional light radiating characteristic. When the dipolar emitter locates at the center of the metastructure (Δ*x* = nm) and without considering the active feature of the gap layer (i.e., *α* = 0 cm^−1^), the far-field radiation shows two resonant peaks, namely, resonance *R*_1_ at 200 THz and *R*_2_ at 235 THz in Fig. 2(a), which actually corresponds to the peak and dip of the Fano-type spectrum in ref. 13, due to constructive and destructive interferences between toroidal and electric dipoles, respectively. It can be further verified by magnetic field distributions of resonance 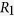 and 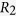 in right panel of Fig. 2. Besides, the calculated scattered powers in terms of multipoles also sufficiently justify the excitation of a dominant toroidal dipole around 200 THz as shown in Fig. 3.

**Figure 4:**
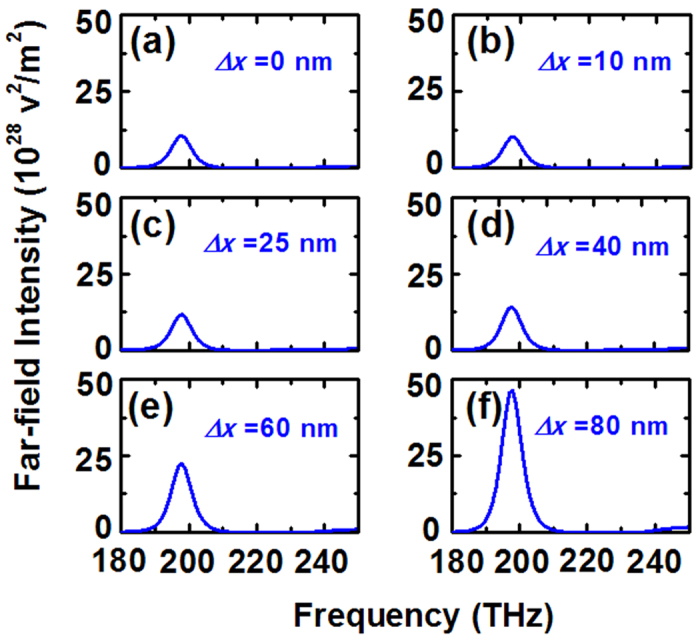
The spectra of far-field intensity monitored by probe D for different radial displacement Δ*x*.

Interestingly, when the position of dipole emitter has a small deviation from the center (e.g., Δ*x* = 10 nm), the far-field radiating intensity of high frequency resonance R_2_ is gradually enhanced. With a continuing shift up to Δ*x* = 80 nm, the far-field radiating power at resonance R_2_ is vastly enhanced to about 264000 times than the ones of Δ*x* = 0 nm at 235 THz, undergoing a transformation from non- to super-radiating and making the emitter radiating like an optical nano-antenna with high directivity. Nevertheless, it should be emphasized that the low frequency resonance R_1_ does not disappear. In order to clarify it, Fig. 4 shows the dependence of the far-field radiating intensity of the resonance R_1_ on different radial displacements, monitored by the probe D: With the dipolar emitter moving along the *x*-axis, the far-field radiating power gradually increased and reached a maximum of 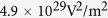 when Δ*x* = 80 nm.

**Figure 5:**
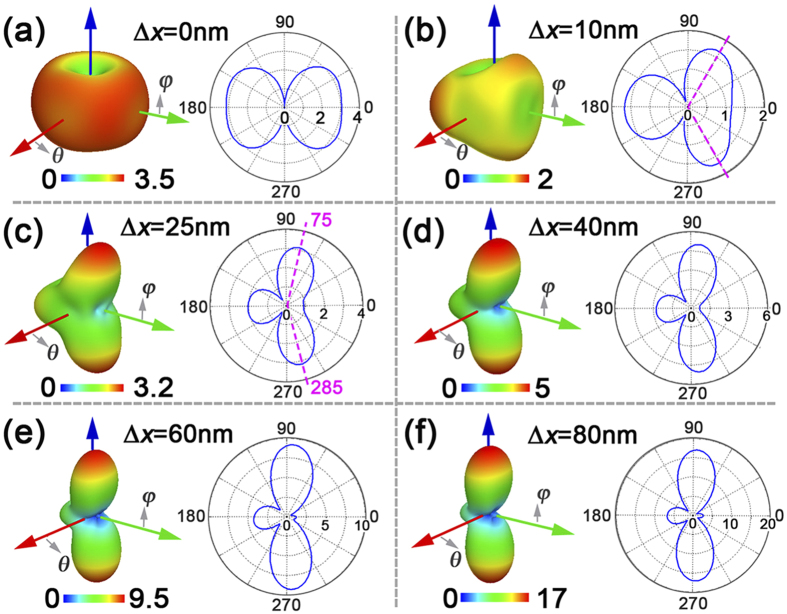
Three-dimensional far-field radiation patterns at 235 THz for different radial displacement Δ*x*, and corresponding two-dimensional patterns in dependence of φ under θ = 90 degree (Radiation unit: 10^8^ V.

To intuitively make clear the radiating evolution process from Δ*x* = 0 nm to Δ*x* = 80 nm, far-field radiating patterns for different radial displacement Δ*x* are presented in Fig. 5. Obviously, before the dipolar emitter moving along the *x*-axis, it radiates light in an azimuthally symmetric torus-like pattern with respect to the *y*-axis. Nevertheless, once there is a small shift of 25 nm, directional radiations occur at 75- and 285-degree orientations (marked by pink dashed-lines) as shown in Fig. 5(c). By further moving to Δ*x* = 80 nm, it will result in a directional radiating parallel to the *y*-axis with an angular width of 60 degree, radiating as an optical nano-antenna. In short, the ultimate physical mechanism is a hybridization interference between toroidal and electric dipoles due to an asymmetric configuration induced by the radial displacement Δ*x*. It is necessary to note that, for the case of Δ*x* = 80 nm, there still leaves a small radiation to the opposite *x*-axis direction on account of an insufficient interference.

**Figure 6:**
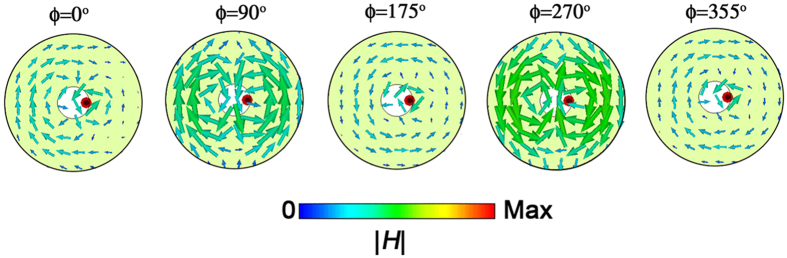
The magnetic field vector distributions correspond to different phases under the geometric configuration of Δ*x* = 80 nm.

Then, Fig. 6 illustrates the magnetic-field distributions at 235 THz to better explain the interference mechanism. In phases 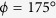 and 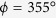, the magnetic field of toroidal dipole response is always out-of-phase to the excitation field from the dipolar emitter. However, for 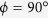 and 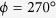, the magnetic field forms a symmetric distribution respect to the *y*-*z* plane, implying a different interference process. Meanwhile, when 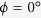, it seems to be a transition state from 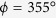 to 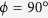. This is to say that the super-radiating characteristic of the single emitter actually results from asymmetric hybridization interference between toroidal and electric dipoles due to a radial displacement of the dipole emitter with respect to the double-flat-ring metastructure.

**Figure 7:**
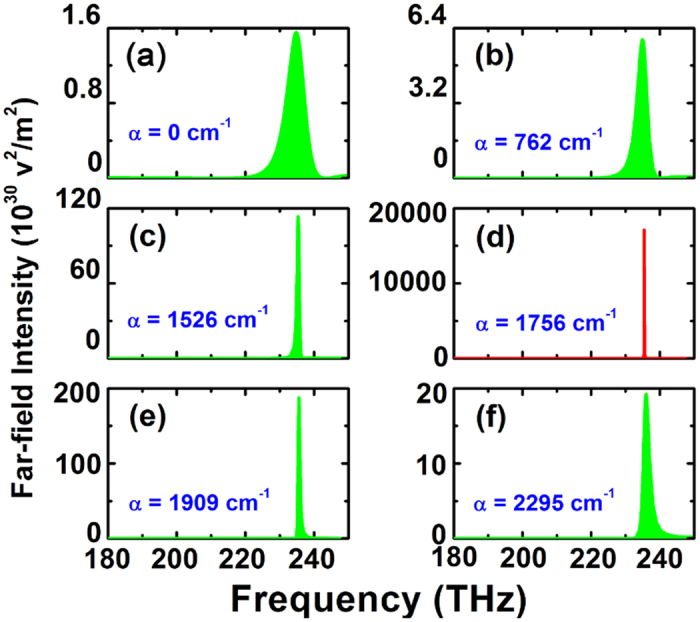
The far-field radiating powers at different gain coefficients for Δ*x* = 80 nm.

**Figure 8:**
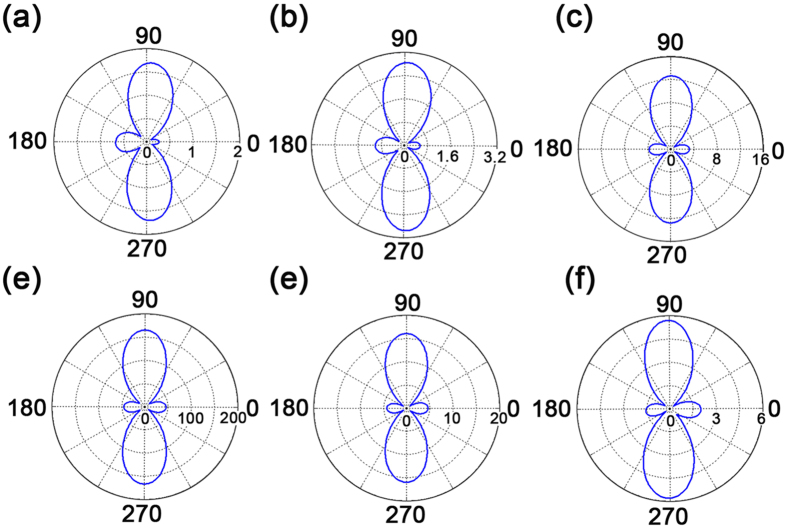
Under the geometric configuration of Δ*x* = 80 nm, the two-dimension radiating patterns (Radiation unit: 10^9^ ) under different gain coefficients at 235 THz. From (**a**) to (**f**), the gain coefficient *α* is 0, 762, 1526, 1756, 1909 and 2295 cm^−1^, respectively.

Since light radiating has great potential applications such as sensing and optical communications, it is of the great importance to enhance the light radiating power and direction of the single dipolar emitter when Δ*x* = 80 nm at 235 THz. Such idea can be achieved by introducing optically active medium into this system. Here, the active medium (for example, PbSe semiconductor quantum dot) is embedded in the silicon-oxide gap layer, as described earlier. In order to provide an intuitive comparison, Fig. 7(a) displays the far-field radiating intensity with a peak of 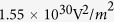 under an absence of gain medium (namely, 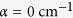). Then, with the gain coefficient *α* increasing, the far-field radiating power is greatly strengthened and obtains a maximum value of 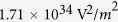 under the moderate gain level of 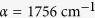, which is 1.1×10^4^ times stronger than ones of 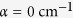. Meanwhile, the sharp enhancement of the far-field radiating power at 235 THz, prompted by the gain medium, directly leads to a smaller full width at half maximum (FWHM), implying the potential for an excellent sensing performance^32^. In addition, the corresponding two dimensional (2D) radiating patterns of different gain values at 235 THz are also presented in Fig. 8. Although the radiating angular width is always 60 degree, it is obvious that the far-field radiating power realizes a tremendous enhancement under a moderate gain level. Nevertheless, it is important to note that the far-field radiation reduction above a certain critical gain coefficient, though in accordance with literatures^33^,^34^, should be attributed to an unrealistic consequence of the time-independent solution. That is to say, if one increases the gain coefficient to beyond certain threshold, the actual physical field will be increasing with time. This contradicts with our numerical calculation based on time-independent Maxwell equations. Therefore, physically there may be no way to obtain such overlarge value of gain coefficient in a steady state^35^.

## Conclusion

In summary, the light radiating of a single emitter can be manipulated by the toroidal metastructure composed of double flat rings, from non- to super-radiating at 235 THz, rooting in hybridization interference between toroidal and electric dipoles due to an asymmetric configuration by shifting the dipole emitter along the radial direction. Gain medium is embedded in the gap layer between top and bottom metallic flat rings to vastly strengthen the far-field super-radiating power. The directive radiation intensity can reach orders of magnitude enhancement with a moderate gain level. It may provide a channel to manipulate the light-matter interaction.

## Additional Information

**How to cite this article:** Li, J. *et al*. Super-radiating manipulation of a nano-emitter by active toroidal metamaterials. *Sci. Rep.* **7**, 46609; doi: 10.1038/srep46609 (2017).

**Publisher's note:** Springer Nature remains neutral with regard to jurisdictional claims in published maps and institutional affiliations.
